# Entwicklung eines Ivermectin-haltigen Saftes als Magistralrezeptur für Kinder zur Therapie der Skabies

**DOI:** 10.1007/s00105-021-04806-4

**Published:** 2021-04-13

**Authors:** Johannes Wohlrab, L. Stadie, R. H. H. Neubert, K. Bosse

**Affiliations:** 1 Universitätsklinik für Dermatologie und Venerologie, Martin-Luther-Universität Halle-Wittenberg, Ernst-Grube-Str. 40, 06120 Halle (Saale), Deutschland; 2 An-Institut für angewandte Dermatopharmazie e. V., Martin-Luther-Universität Halle-Wittenberg, Halle (Saale), Deutschland

**Keywords:** Orale Applikation, Kindesalter, Off-label-Anwendung, Körpergewichtsadaptierte Dosierung, Applikationshilfen, Oral administration, Children, Off-label use, Body-weight-adapted dosage, Delivery aids

## Abstract

**Hintergrund:**

Zur Therapie der Skabies kann orales Ivermectin eingesetzt werden. Die Evidenz für einen sicheren und wirksamen Einsatz bei Kleinkindern im Einzelheilversuch ist erarbeitet worden und publiziert. Um eine körpergewichtsadaptierte Dosierung auch für Kinder zu gewährleisten, wurde ein Ivermectin-haltiger Saft als Magistralrezeptur entwickelt.

**Material und Methoden:**

Da Ivermectin nicht als Reinsubstanz für die Rezeptur zur Verfügung steht, wurden wirkstoffhaltige Tabletten als Ausgangsmaterial für die Entwicklung benutzt. Die Formulierung wurde entsprechend pharmazeutischer, regulatorischer und gebrauchsorientierter Kriterien konzipiert. Zum Nachweis der chemischen Stabilität wurde eine HPLC(Hochleistungsflüssigkeitschromatographie)-Methode erarbeitet und validiert. Um die praktische Umsetzung zu erleichtern, wurden zudem Angaben zu geeigneten Packmitteln und zu Applikationshilfen erarbeitet, und die Rezeptur wurde taxiert.

**Ergebnisse:**

Es konnte nachgewiesen werden, dass die finale Rezeptur stabil in der Apotheke hergestellt und über 3 Wochen gelagert werden kann. Es haben sich keine Bedenken bezüglich der Verträglichkeit des Rezeptursaftes ergeben. Die physikochemischen Eigenschaften und der Geschmack der Rezeptur ermöglichen den beabsichtigten Gebrauch als gut dosierbaren Saft für Kinder.

**Schlussfolgerung:**

Die entwickelte Rezeptur entspricht den Anforderungen der Apothekenbetriebsordnung (§ 7 ApBetrO) und ermöglicht eine exakte, körpergewichtsadaptierte Dosierung von oralem Ivermectin bei Kleinkindern. Untersuchungen zur Pharmakokinetik am Menschen bzw. klinische Studien zum Nachweis der Verträglichkeit und/oder Wirksamkeit liegen für die Rezeptur nicht vor.

## Abstract (en)

**Background:**

Oral ivermectin can be used to treat scabies. Evidence for safe and effective use in young children in individual treatment situations has been developed and published. In order to also ensure a body weight-adapted dosage for children, an ivermectin-containing syrup was developed as an extemporaneous preparation.

**Materials and methods:**

Since ivermectin is not available as a pure substance for the formulation, tablets containing active ingredient were used as a basic material for development. The formulation was designed according to pharmaceutical, regulatory and use-oriented criteria. An HPLC (high-pressure liquid chromatography) method was developed and validated to demonstrate chemical stability. In order to facilitate the practical implementation, information on suitable packaging material and application aids was also developed and the formulation was evaluated.

**Results:**

It has been demonstrated that the final formulation produced in the pharmacy was stable and can be stored for 3 weeks. No concerns were raised regarding the tolerability of the syrup formulation. The physicochemical properties and the taste of the formulation allow the intended use as a well-dosed syrup for children.

**Conclusion:**

The developed formulation meets the requirements of the Apothekenbetriebsordnung (Pharmacy Work Rules; Section 7 ApBetrO) and enables an exact, body weight-adapted dosage of oral ivermectin in young children. Studies on human pharmacokinetics or clinical studies to demonstrate tolerability and/or efficacy are not available for the formulation.

Für die Behandlung einer Skabiesinfestation stehen verschiedene Arzneistoffe zur Verfügung. Neben topischen Therapieoptionen mit Permethrin (5 %), Benzoylbenzoat (10–25 %) oder Crotamiton (5–10 %) kann bei gewöhnlicher Skabies die orale Applikation von Ivermectin als Standard der Zweitlinientherapie gelten [1, 2]. Bei immunsupprimierten Patienten, starken entzündlichen oder erosiven Begleitsymptomen bzw. bei milbenreichen Formen wird orales Ivermectin auch in der Erstlinientherapie eingesetzt [1]. Wegen der zunehmenden Verbreitung und dem damit gehäuften Auftreten der Skabies mehren sich Verdachtsmomente für eine unzureichende Wirksamkeit topischer Therapien, insbesondere bei der Verwendung von Permethrin. So findet sich eine zunehmende Zahl von Patienten, die trotz wiederholter topischer Behandlung und propagierter Umfeldsanierung nachweislich Skabiesrezidive entwickeln. Die Ursachen hierfür können vielfältig sein [3]. Dabei ist v. a. an eine fehlerhafte Anwendung der topischen Therapie zu denken, allerdings müssen auch Rezidive durch unerkannte Infestationsquellen im Umfeld des Patienten sowie möglicherweise Resistenzentwicklungen auf Permethrin bedacht werden [3–5]. Vor diesem Hintergrund hat sich bei Patienten mit Rezidivskabies die Mono- oder Kombinationstherapie mit oralem Ivermectin etabliert [6]. Als Derivat der Avermectine entfaltet Ivermectin seine Wirkung durch Blockade des Neurotransmitters Gamma-Aminobuttersäure (GABA) und interagiert zudem direkt mit Glycin‑, Histamin- und Nikotinacetylcholinrezeptoren [7]. Durch Bindung an glutamatgesteuerte Chloridkanäle in Nerven und Muskeln führt es zur Depolarisation und damit zu Lähmung sowie Absterben von Skabiesmilben [8]. Ivermectin ist für die Therapie der Skabies für Erwachsene sowie Kinder ab einem Körpergewicht von 15 kg in einer Dosierung von 200 µg/kg Körpergewicht zugelassen. Mittlerweile liegen aber auch umfangreiche Daten für die sichere und wirksame Anwendung von Ivermectin bei Kindern unter 15 kg Körpergewicht vor, sodass eine Anwendung bei wiederholt unwirksamer topischer Therapie als Einzelheilversuch vertretbar erscheint [9–13]. Da Ivermectin als Rezeptursubstanz nicht in Arzneibuchqualität verfügbar ist, muss auf ein Fertigarzneimittel in Form von 3 mg Tabletten zurückgegriffen werden, was eine exakte, körpergewichtsadaptierte Dosierung problematisch gestaltet. Vor diesem Hintergrund wurde ein Ivermectin-haltiger Saft für Kinder entwickelt, der aus einem Fertigarzneimittel hergestellt werden kann und hinsichtlich Stabilität, Konsistenz und Geschmack dem beabsichtigten Gebrauch angepasst wurde.

## Material und Methoden

### Vehikelkonzeption

Zur Erarbeitung eines galenischen Konzeptes wurden die Inhaltstoffe des Fertigpräparates Scabioral® (InfectoPharm Arzneimittel und Consilium GmbH, Heppenheim, Deutschland) bzw. alternativ des aus gleicher Produktion stammenden Generikums Driponin® (Pädia GmbH, Heppenheim, Deutschland) analysiert. Durch theoretische Erwägungen, die sich aus den physikochemischen Eigenschaften des Wirkstoffs bzw. der im Fertigpräparat enthaltenen Hilfsstoffe (mikrokristalline Zellulose, vorverkleisterte Maisstärke, Butylhydroxyanisol und Magnesiumstearat) sowie den Besonderheiten der Pharmakokinetik im Kindesalter ergeben, wurde für die Entwicklung eines Saftes eine Öl-in-Wasser(O/W)-Emulsion favorisiert. Die Auswahl notwendiger zusätzlicher Hilfsstoffe erfolgt v. a. auf der Basis regulatorischer (z. B. Sicherheit, Zulassung, Verfügbarkeit, Qualität, Eignung, Preis) sowie pharmazeutischer Erfordernisse (z. B. Stabilität, Viskosität, Geschmack). Für die Herstellung einer stabilen Emulsion wurden als Emulgator Polysorbat 60 und zur Einstellung der Viskosität der Rezeptur als Gelbildner Carmellose-Natrium ausgewählt. Die Anpassung der Geschmackseigenschaften wurde durch Zusatz von Süß- (Sorbitol 70, Saccharin-Natrium) und Aromastoffen (Himbeeraroma) eingestellt. Dabei wurde auf eine angenehme Süße und das Vermeiden eines bitteren sowie unangenehmen Bei- oder Nachgeschmacks geachtet. Zur pH-Einstellung kam Zitronensäure zum Einsatz. Zur mikrobiologischen Stabilisierung wurde die Rezeptur mit Natriumsorbat konserviert. Alle verwendeten Hilfsstoffe sind als Rezeptursubstanzen für Apotheker in Arzneibuchqualität verfügbar (WEPA Apothekenbedarf GmbH & Co KG, Hillscheid, Deutschland bzw. Caesar & Loretz GmbH, Hilden, Deutschland).

### Löslichkeitsverhalten von Ivermectin

Ivermectin besitzt einen logP-Wert von 4,1 und ist somit gut fett-, aber nicht wasserlöslich. Durch eine systemische Bioverfügbarkeit von 50–60 % ergibt sich für diesen Stoff gemäß Biopharmaceutical Classification System (BCS) deshalb die Einordnung in die BCS-Klasse IV [14, 15]. Durch die Anwendung von Ivermectin in gelöster Form lässt sich dessen systemische Bioverfügbarkeit deutlich erhöhen [16]. Deshalb wurde das Löslichkeitsverhalten des Arzneistoffs in relevanten Ölen untersucht, um eine Verarbeitung in gelöster Form auch gewährleisten, gleichzeitig aber auch die Quantität der Ölphase der Formulierung so gestalten zu können, dass die Stabilität der Emulsion erhalten bleibt. Dazu wurde das mittlere Tablettengewicht des Fertigarzneimittels bestimmt, um die äquivalente Masse einer gemörserten Tablette entsprechend 1 mg Ivermectin exakt zu bestimmen. Die so ermittelte Masse des Mörsepulvers wurde schrittweise in einer aufsteigenden Menge Rapsöl von 0,1–0,7 g in 0,1-g-Schritten dispergiert. Jeweils 20 mg des Mörsepulver-Öl-Gemisches wurden in 1,8 ml Methanol extrahiert, anschließend auf 10 µg/ml verdünnt und der Ivermectin-Gehalt mittels HPLC (Hochleistungsflüssigkeitschromatographie) vermessen.

### Vehikelcharakterisierung

Zur Charakterisierung der Rezeptur wurden Spreitbarkeit, Viskosität, pH-Eigenschaften, Brechungsindex und Dichte des Vehikels bestimmt. Die Spreitbarkeit beruht auf den rheologischen Eigenschaften einer Matrix und ermöglicht Aussagen zur Konsistenz. Hierfür wurde in eine, mit luftblasenfreier Formulierung gefüllte Dosierplatte des Extensometers (Apotec, WEPA Apothekenbedarf GmbH & Co. KG, Hillscheid, Deutschland) ein 4‑mm-Stempel eingedrückt und nach Entfernen von überschüssigem Material mit einem Kartenblatt eine skalierte Messplatte von oben mittig horizontal platziert. Nach einer Konsolidierungszeit von 1 min wurde der Radius der Spreitungsfläche mithilfe der Messskala in 4 Richtungen rechtwinklig zum Skalenverlauf abgelesen. Aus den Mittelwerten der Ausbreitungsradien wurde die Spreitbarkeit in mm^2^ errechnet. Die Viskosität wurde mittels Viskosimeter (Rotavisc hi-vi I, IKA Labortechnik, Staufen, Deutschland) bei konstanter Temperatur (20 °C Raumtemperatur) und steigender Drehzahl (0–200 rpm) vermessen. Dabei wurde die Drehzahl jeweils für 1 min gehalten, und die gemessenen Viskositäten wurden gemittelt. Die Mittelwerte wurden gegen die sich ergebene Schubspannung aufgetragen. Durch ein mit dem Viskosimeter verbundenes Thermostat (HRC 2 basic, IKA Labortechnik, Staufen, Deutschland) wurde zudem die Viskosität in Abhängigkeit von der Temperatur gemessen. Hierzu wurde die Rezeptur jeweils bei Raumtemperatur (20 und 7 °C) mit einer konstanten Drehzahl von 35 rpm in 3 verschiedenen Konzentrationen für 5 min vermessen (*n* = 4). Die Datenauswertung erfolgte mit der Software Labworldsoft® 6 Pro (IKA Labortechnik, Staufen, Deutschland).

Die Validierung der pH-Eigenschaften erfolgte mittels pH-Meter (FiveEasy FE20, Mettler Toledo, Gießen, Deutschland), das vor den Messungen in einer Zweipunktmethode mit eingestellten Pufferlösungen bei pH 4,01 und pH 7,00 kalibriert wurde. Der Ziel-pH-Wert wurde entsprechend physikochemischen Gegebenheiten, Stabilitäten der Inhaltstoffe und Verträglichkeit der Rezeptur als Oralia im Bereich zwischen pH 3 und 5 definiert. Der Brechungsindex als Stoffeigenschaft der Rezepturmatrix wurde mittels Refraktometer (RM40, Mettler Toledo, Gießen, Deutschland) bei 25 °C ermittelt. Zur exakten Dosierung der Rezeptur wurde die Zusammensetzung in Masseprozent (m/m) erarbeitet und die Dichte mittels Pyknometer nach Gay-Lussac bestimmt. Dazu wurde das leere Pyknometer gewogen, anschließend luftblasenfrei mit der Rezepturmatrix gefüllt und erneut gewogen. Die Massedifferenz wurde zur Berechnung der Dichte herangezogen.

### Auswahl eines geeigneten Packmittels und Applikators

Die chemische Stabilität von Ivermectin kann durch photochemische Reaktionen beeinträchtigt werden. Deshalb muss ein eingesetztes Packmittel Lichtschutz gewährleisten. Die für die Applikation niedrigvisköser Oralia übliche Dosiervorrichtung wie Dosierlöffel oder Becher ist wegen einer unzureichenden Dosiergenauigkeit für den beabsichtigten Einsatz der vorliegenden Rezeptur ungeeignet. Deshalb wurde eine Dosierspritze favorisiert. Bei der Auswahl und Erprobung von potenziellen Packmitteln wurde das an die Anwendungsgegebenheiten adaptierte Volumen der Rezeptur zugrunde gelegt. Dabei ergab sich für die Verarbeitung einer Tablette, enthaltend 3 mg Ivermectin, bei einer Zielkonzentration von 1 mg/ml ein Rezepturvolumen von 3 ml bzw. bei einer Zielkonzentration von 400 µg/ml ein Rezepturvolumen von 7,5 ml. Deshalb wurde eine Flasche mit einem Volumen von 10 ml mit passendem Spritzenaufsatz und geeigneten Spritzen (1 ml bzw. 5 ml Kolbenpipetten) als Packmittel favorisiert.

### Erarbeitung und Validierung einer analytischen Methode

Zur Ermittlung der Wirkstoffstabilität wurde eine HPLC-Methode (Agilent Technologies 1290 Infinity LC, Waldbronn, Deutschland) entwickelt. Die Methodenentwicklung und -validierung erfolgten nach den Vorgaben internationaler Standards (USP36 NF31) und der USP(United States Pharmacopeia)-Monographie für Ivermectin [17]. Zur Methodenentwicklung wurden 2 unterschiedliche Säulen (Nucleosil 120‑3 C18 125 × 2 mm und Nucleodur 100‑3 C18 ec, 150 × 4 mm, Macgerey-Nagel, München, Deutschland) und verschiedene Verhältnisvariationen eines Lösungsmittelgemisches aus Methanol, Wasser und Ameisensäure ausgetestet. Die Validierung der Methode erfolgte mittels quantitativer Analyse mit externen Standards (Kalibrierstandards [Cals], Qualitätskontrollstandards [QCs]), die an 3 voneinander unabhängigen Tagen (3-Tage-Validierung) vermessen wurden. Dadurch konnten die Richtigkeit („within-run-accuracy“, „between-run-accuracy“), Präzision („carry-over“, „lower limit of quantification“ (LLOQ), Selektivität) und Robustheit der Methode untersucht werden [18–22].

### Stabilitätsuntersuchungen

Die Stabilität der Formulierungen wurde auf chemischer, organoleptischer und mikrobiologischer Ebene untersucht. Zur Bestimmung der Gleichförmigkeit des Gehalts wurden Untersuchungen in Anlehnung an die Testung einzeldosierter Arzneiformen nach European Pharmacopoeia (Ph. Eur.) 2.9.6 und 2.9.40 durchgeführt [23]. Den Ivermectin-haltigen Formulierungen wurden jeweils 3‑mal 1 ml entnommen und davon jeweils 10 äquivolume Aliquote hergestellt. Aus jeder Einzelprobe wurde Ivermectin mit Methanol extrahiert und der Gehalt bestimmt. Diese Prozedur wurde für 3 unabhängige Chargen jeder Herstellungsvariante (Fanta-Schale und Pistill mit 5 min Rührzeit, Fanta-Schale und Pistill mit Homogenisierung über Wasserbad [40 °C] und Kaltrühren, Homogenisierung mit Ultraturrax [IKA T18 basic, IKA Labortechnik, Staufen, Deutschland]) durchgeführt. Zur Bestimmung der Langzeitstabilität wurden die Formulierungen in 5‑ml-Einmalspritzen abgefüllt und im Brutschrank bei 40 °C für 5 Wochen gelagert. Dabei wurde der Füllstand auf der Spritze markiert. In der ersten Woche wurden täglich 3 Proben pro Formulierung entnommen und bezüglich Farbe, Geruch, pH-Wert, Auskristallisation, Brechungsindex, Gewicht, Homogenität, Spreitbarkeit, Phasentrennung und Gehalt überprüft. Anschließend erfolgte die Entnahme von jeweils 3 Proben nach 3 und 5 Wochen [24]. Die Ausgangswerte der Parameter wurden vor der Einlagerung der Gefäße bestimmt und dienten als Referenzwerte. Die mikrobielle Stabilität der Formulierungen wurde mittels Zählung der vermehrungsfähigen Mikroorganismen nach Ph. Eur. 2.6.12. validiert [23]. Hierbei wurden die Gesamtkeimzahl („total aerobic microbial count“ [TAMC]) sowie die Anzahl von Hefen und Schimmelpilzen („total combined yeasts/moulds count“ [TYMC]) durch das Auszählen auf Agarplatten bestimmt.

### Präklinische Validierung der Schleimhautverträglichkeit

Die Schleimhautverträglichkeit wurde im Hühnereimodell an der Chorion-Allantois-Membran (CAM) (HET-CAM-Test) überprüft [25, 26]. Dazu kam das Verfahren entsprechend internationaler Standards zum Einsatz [27]. Es wurden natürlich befruchtete Hühnereier (50–60 g) der Rasse „New Hampshire“ verwendet, die über 8 Tage bei 37 °C und 55 % Luftfeuchtigkeit in Paletten in einem Brutschrank bebrütet, dann eröffnet und präpariert wurden. Zur Beurteilung jeder Formulierung wurden an jeweils 6 Eiern maximal 30 min nach dem Eröffnen je 300 mg der Testpräparation appliziert. Zur Quantifizierung der Verträglichkeit wurden mittels eines visuellen Scores die Toxizitätsparameter der CAM nach Interagency Coordinating Committee on the Validation of Alternative Methods (ICCVAM) bestimmt [27].

### Taxierung der Rezeptur

Zur Taxierung der Rezeptur wurden die Apothekeneinkaufspreise für die jeweils verfügbare kleinste Abgabemenge der Bestandteile und Packmittel recherchiert und zusammen mit den Herstellungskosten entsprechend der Apothekenbetriebsordnung kalkuliert. Durch Variationen des Bezugs und damit unterschiedlicher Preise sind Abweichungen der hier berechneten Kosten möglich.

## Ergebnisse

Aus praktischen Gründen wird die Ergebnispräsentation im Folgenden auf die relevanten Inhalte der letztlich ausgewählten Formulierung fokussiert und auf die Darstellungen von Daten zu weiteren 24 Vehikelvarianten verzichtet.

### Löslichkeitsverhalten

Als theoretische Zielkonzentration des Ivermectin in der Ölphase wurden 11 µg/ml im Extraktionsmittel Methanol definiert. In Tab. 1 sind die Ergebnisse der Löslichkeitsuntersuchungen wiedergegeben. Dabei zeigt sich insbesondere bei 0,2 g Öl eine große Abweichung vom Zielwert, die durch die ungleichmäßige Verteilung des Wirkstoffs im Mörsepulver erklärt werden kann. Dennoch zeigen die Daten, dass ab einer Ölmenge von 0,1 g, sicher ab 0,3 g die Masse von 1 mg Ivermectin gut löslich ist. Dies entspricht für 1 Tablette mit 3 mg Ivermectin einer Mindestmenge von 0,5 g Öl. Gleichzeitig verdeutlichen diese Resultate, dass eine Teilung von Tabletten ein hohes Risiko einer ungenauen Dosierung beinhaltet.

**Table 1:** 

Rapsöl (g)	0,1	0,2	0,3	0,4	0,5	0,6	0,7
Ivermectin (μg/ml)	8,818	4,696	10,241	9,458	10,443	10,928	10,928
	8,811	4,708	10,236	9,466	10,452	10,955	10,955
	8,842	4,710	10,136	9,510	10,515	10,878	10,878

### Charakterisierung

**Figure 1:**
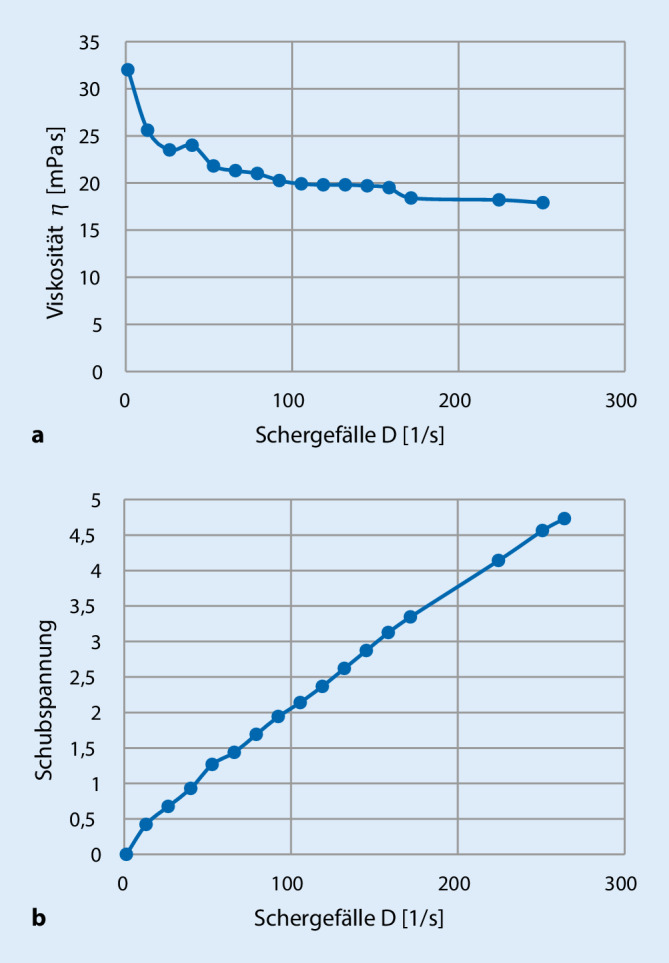

Bezüglich der Konsistenz kann festgestellt werden, dass mit zunehmendem Emulgator‑, Sorbitol- und Gelbildneranteil die Viskosität erwartungsgemäß zunimmt. Für die Herstellung der Rezeptur ist es weiterhin bedeutsam, dass die Zugabe der Ölphase sowie die Hilfsstoffe der Tablette die Viskosität erhöhen, sodass die Gelgrundlage eine sehr niedrige Viskosität hat. Zur Charakterisierung der rheologischen Eigenschaften der Rezeptur wurden ein Viskosigramm und ein Rheogramm (Abb. 1) erstellt. Die Ergebnisse lassen auf ein pseudoplastisches Fließverhalten schließen. Dabei zeigt sich ein für Gele typisches rheologisches Verhalten einer Scherverdünnung (Thixotropie), also ein Absinken der Viskosität bei steigender Scherbeanspruchung. Dieses thixotrope Verhalten ist gewünscht, da sich durch Schütteln der Zubereitung die Fließeigenschaften und damit die Dosiereigenschaften verbessern. Die Daten zeigen zudem, dass durch Abkühlung der Formulierung von 20 auf 7 °C die Viskosität zunimmt (Tab. 2). Somit ergibt sich bei Zimmertemperatur eine geeignet niedrige Viskosität von Fruchtsaftkonsistenz, die eine gute Dosierbarkeit gewährleistet (Vergleichswerte: Wasser ca. 1 mPa s, Fruchtsaft ca. 50 mPa, Olivenöl ca. 100 mPa). Der pH-Wert der Formulierung wurde auf 3,8 bis 3,9 eingestellt, sodass der Bereich von 3,0 bis 5 stabil erreicht wird. Damit sind sowohl die Stabilität der freien Glykolsäure des Carmellose-Natriums (pH > 3,0) als auch die Wirkung von Kaliumsorbat (pH < 6,0) und die Entfaltung der Fruchtaromen (pH < 5,0) gewährleistet. Zur Geschmacksoptimierung wurde die Emulgatorkonzentration auf 2 % begrenzt. Wegen des unangenehmen Eigengeschmacks des verwendeten Öls wurde die Aromakonzentration auf 0,5 % festgelegt, sodass insgesamt ein runder, angenehmer Geschmack resultiert. Die Dichtemessung der wirkstofffreien Formulierung ergab für 4 unabhängig hergestellte Chargen einen Wert von 1,0672 g/ml, sodass der Qualitätskontrolle in der Apotheke näherungsweise ein Wert von 1,0 g/ml zugrunde gelegt werden kann.

**Table 2:** 

	Viskosität (mPa s ± SD)	
	7 ± 0,2 °C	20 ± 0,3 °C
Placebo	38,85 ± 4,57	23,75 ± 2,59
400 μg/ml	73,63 ± 4,04	39,78 ± 1,77
1 mg/ml	108,33 ± 9,49	63,58 ± 2,75

### Auswahl eines geeigneten Packmittels mit Applikator

Durch die Analyse möglicher Packmittel wurden Primärpackmittel identifiziert, die in der Apotheke verfügbar sind, die Stabilität der Formulierung gewährleisten und die Nutzung eines Applikators zur exakten Dosierung ermöglichen (Tab. 3).

**Table 3:** 

	Genaue Bezeichnung	Anbieter	Artikel	PZN
Primärpackmittel	Glasflasche GL 18 10 ml	WEPA, Hillscheid, Deutschland	032631	–
	Tropfflasche 10 ml	Caelo, Hilden, Deutschland	1001	10189381
	Tropfflasche 20 ml	Caelo	1002	10189398
Schraubdeckel mit Pipetteneinsatz	+ Schraubmontur weiß mit Steckeinsatz GL 18 mit KISI und OV für Kolbendosierpipette	WEPA	032403	01445945
Kolbendosierpipette	Kolbendosierpipette 1 ml (geteilt in 0,05 ml)	WEPA	032401	01442970
	Kolbendosierpipette 5 ml (geteilt in 0,2 ml)	WEPA	032402	01445862
	Dronabinol-Tropfen-Dosierspritze	Caelo	1059	15257259

*PZN* Pharmazentralnummer, *KISI* Kindersicherheitsverschluss, *OV* Originalitätssicherungverschluss

### Analytische Methode

Da Ivermectin eine sehr lipophile Substanz darstellt, wurden der in der USP-Methode hohe hydrophile Anteil des Laufmittels und damit die Laufzeit reduziert. Dabei wurde auf eine klare Trennung des Wirkstoff- vom Einspritzpeak geachtet. Als Lösungsmittelgemisch wurde Ameisensäure zur Protonierung des Ivermectin eingesetzt und Acetonitril durch Methanol wegen der höheren Selektivität auf hydrophilen Säulen ausgetauscht. Zudem wurde eine Vorsäule eingesetzt, um Verunreinigungen der Analysesäule zu vermeiden. In Tab. 4 sind die HPLC-Methoden aus USP und die erarbeitete gegenübergestellt. Durch die Optimierung der Methode und Austausch der Säule konnte zudem eine Reduktion der Laufzeit von 40 auf 5,5 min erzielt werden. Die Methodenvalidierung unter Berücksichtigung der Parameter Präzision, Richtigkeit, Selektivität, Nachweis- und Bestimmungsgrenze, „carry-over“ und Linearität wurden durch Kalibrierung ermittelt. Dabei konnte die Selektivität der Methode für Ivermectin gegenüber den Tablettierhilfsstoffen und den Gelbestandteilen dargestellt werden. Mit einem Regressionskoeffizienten von R > 0,99 in allen Untersuchungen weist die Kalibrierkurve eine gute Linearität auf. Die relativen Fehler und Variationskoeffizienten der Kalibrier- und Qualitätskontrollproben wichen um maximal 2,5 % bzw. 7,3 % vom Soll-Wert ab und erfüllten damit die Voraussetzungen für die Richtigkeit der Methode. Durch Messungen von Blindproben konnte zudem ein „carry-over“ ausgeschlossen werden. Die Nachweis- und Bestimmungsgrenze wurde durch das Signal-Rausch-Verhältnis der Methode ermittelt. Die Nachweisgrenze kann somit mit 0,046 µg/ml und die Bestimmungsgrenze mit 0,220 µg/ml angegeben werden.

**Table 4:** 

	USP	Neue Methode
Säule	250 × 4,6 mm (L1 RP-18)	Nucleodur 100‑3 C18 ec, 150 × 4 mm + Universalvorsäule EC 3/4
Mobile Phase	Acetonitril: MeOH: H_2_O 106:55:39 (V/V/V)	MeOH: H_2_O: Ameisensäure 93:7:0,1 (V/V/V)
Retentionszeit	Ca. 40 min	5,5 min
Druck	118 bar	142 bar
Flussrate	1,5 ml/min	0,8 ml/min
Injektionsvolumen	10 μl	
Detektion	UV: 245 nm	
Lösungsmittel	MeOH	

*USP* United States Pharmacopeia, *MeOH* Methanol, *H*_*2*_*O* Wasser

### Stabilitätsuntersuchungen

Um die Gleichförmigkeit der Formulierung sicherzustellen, wurde eine Akzeptanzwertberechnung gemäß Ph. Eur. 2.9.40 nach der Formel: $AV=\left| M-\overline{X}\right| +ks$ zugrunde gelegt [23]. Dabei ergab sich für eine Konzentration von 400 µg/ml ein Akzeptanzwert von 0,47 und für eine Konzentration von 1,0 mg/ml ein Akzeptanzwert von 27,82. Um die außerhalb der Akzeptanzgrenzen liegende Formulierung mit 1,0 mg/ml Ivermectin besser bewerten zu können, wurde eine Fehlersuche durchgeführt. Dazu wurde der Gehalt in einem größeren Probenvolumen (0,2 ml) und anschließend wurden weitere Proben der unterschiedlichen Herstellungsmethoden vergleichend gemessen. Hier konnte keine Formulierungsvariante mit einer Konzentration von 1 mg/ml identifiziert werden, die den definierten Akzeptanzwert erreichte. Im Schaukeltest hat sich herausgestellt, dass wegen des hohen Anteils der Formulierung an Zuckeralkohol die Gefahr der Abtrennung des vergleichsweise hohen Sorbitolanteils besteht. Andererseits bedingt ein zur Optimierung des Fließverhaltens notwendig niedriger Polysorbat- und Carmelloseanteil eine Phasentrennung. Dies macht es notwendig, dass die Formulierung vor Anwendung geschüttelt wird, um eine vollständige Redispersion zu gewährleisten. Die Untersuchungen zur Langzeitstabilität ergaben am Tag 21 Abweichungen im Brechungsindex von −0,0023, im pH-Wert von −0,02 und nach 21 Tagen im Wirkstoffgehalt von −4,87 %, sodass die jeweiligen Grenzwerte erfüllt wurden. Damit kann die Stabilität nach Lagerung über 3 Wochen belegt werden. Die mikrobiologische Stabilität entsprechend der Keimzahlbestimmung entsprechend Ph. Eur. 6.0 zeigte für die Formulierung in beiden untersuchten Konzentrationen (400 µg/ml und 1,0 mg/ml) sowohl frisch nach Herstellung als auch nach Lagerung über 5 Wochen bei 40 °C keine Einschränkungen der mikrobiologischen Stabilität.

### Präklinische Untersuchungen zur Sicherheit

Die Untersuchungen wurden gemäß Standards referenzkontrolliert durchgeführt und haben keine Hinweise oder Verdachtsmomente für ein irritatives Potenzial der Formulierung ergeben.

### Taxierung und Angaben zur Herstellung

Die Herstellung erfolgt in 3 Schritten:

- Herstellung der Gelgrundlage,
- Herstellung einer öligen Ivermectin-Lösung und
- Herstellung der Endformulierung durch Vermischen der beiden Matrices.

**Figure 2:**
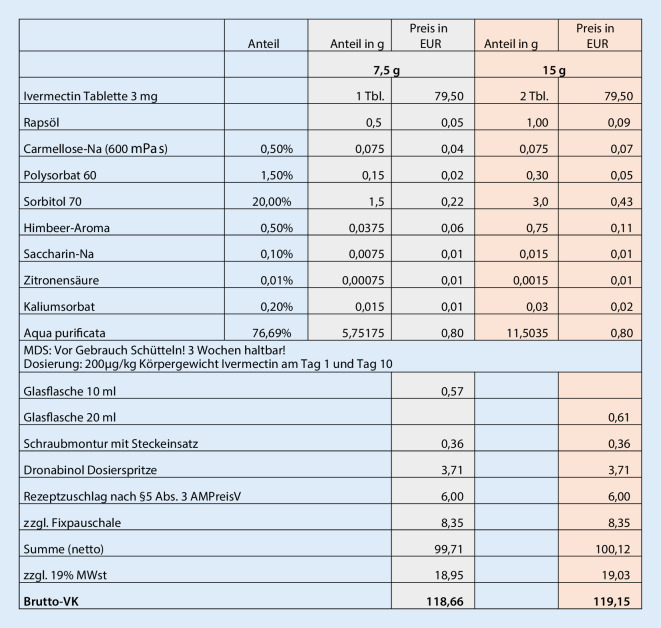

Für die Herstellung der Gelgrundlage sollte ein Becherglas mit Glasstab tariert und die abgewogene Menge Polysorbat 60 mit Wasser gelöst werden. Anschließend erfolgt die Zugabe der abgewogenen Mengen des bereits gelösten Kaliumsorbates und anschließend der Zitronensäure. Schließlich erfolgt die Zugabe der restlichen Inhaltstoffe unter Rühren bis ein klares, homogenes Gel entsteht. Zur Herstellung der öligen Wirkstofflösung wird eine Tablette 3 mg Ivermectin in einer Fanta-Schale vorgelegt und mit 0,5 ml Rapsöl ergänzt. Anschließend wird die Tablette mit dem Pistill zerdrückt und im Öl angerieben. Die Endformulierung wird nun durch Zugabe der abgewogenen Menge Gelgrundlage in die Fanta-Schale mit der öligen Wirkstofflösung und der Homogenisierung der Phasen mit dem Pistill hergestellt und schließlich in das Primärpackmittel umgefüllt und verschlossen. Als Abgabepreis der Rezeptur wurden ca. 120,00 € taxiert. Der Betrag ist als Orientierung zu verstehen, und der reale Preis kann in geringem Umfang abweichen. Die Taxierung für die Ordination von „Ivermectin-Saft 400 µg/ml für Kinder“ ergibt sich aus den Angaben der Abb. 2, und die berechnete Dosierung des Saftes entsprechend dem Körpergewicht sowie die notwendige Rezepturmenge für eine 2‑malige Applikation ergeben sich aus Tab. 5. Die Herstellungsvorschrift für Apotheker mit exakten Angaben zur Plausibilitätsprüfung und Herstellung der Rezeptur kann über die E‑Mail-Adresse „rezeptur@iadp.eu“ angefordert werden.

**Table 5:** 

Körpergewicht (kg)	Zu applizierende Menge (ml)	Herzustellende Menge für 2 Applikationen (g)
5	2,5	7,5
6	3	7,5
7	3,5	7,5
8	4	15
9	4,5	15
10	5	15
11	5,5	15
12	6	15
13	6,5	15
14	7	15

## Diskussion

Die Ergebnisse zeigen, dass es gelungen ist, auf der Basis des verfügbaren Fertigarzneimittels in Form von Ivermectin-haltigen Tabletten eine den regulatorischen Erfordernissen entsprechende standardisierte Rezeptur zu erarbeiten. Aus der Literatur zur Pharmakologie von Ivermectin können weitgehende Rückschlüsse auf potenzielle Risiken bei der Verwendung der Rezeptur abgeleitet werden. Diese ergeben sich v. a. aus einer möglicherweise erhöhten systemischen Bioverfügbarkeit von Ivermectin durch die Lösung des Wirkstoffs in der Rezepturmatrix [16]. Im Extremfall wäre hier eine um ca. 10–20 % höhere Bioverfügbarkeit denkbar, sodass die Applikationsdosis von 200 µg/kg Körpergewicht nicht überschritten werden sollte. Dies ist durch die exakte Dosiermöglichkeit mittels der ausgewählten Applikationshilfe im Gegensatz zum Fertigarzneimittel auch gewährleistet. Bei Beachtung dieser Dosierempfehlung ist eine toxikologisch relevante erhöhte Bioverfügbarkeit weitgehend auszuschließen. Aufgrund der Stabilitätsdaten ist die Rezeptur mit einer Konzentration von 400 µg/ml klar zu präferieren. Diese erfüllt nicht nur ausnahmslos alle Kriterien einer standardisierten Rezeptur, sondern ermöglicht auch eine exakte Dosierung und wird damit allen Erfordernissen der praktischen Anwendung gerecht. Dennoch muss festgehalten werden, dass für die entwickelte Rezeptur bisher keine pharmakokinetischen Daten am Menschen bzw. Daten zur Verträglichkeit und/oder Wirksamkeit vorliegen. Die Bewertung der Pharmakokinetik und Sicherheit der Rezeptur ergibt sich aus tierexperimentellen Daten sowie aus Beobachtungsstudien an Kindern unter 15 kg Körpergewicht, die in der Indikation Onchozerkose bzw. Skabies mit systemischem Ivermectin behandelt wurden [9–13]. Der Einsatz der Magistralrezeptur sollte deshalb mit der gebotenen Vorsicht, nur bei klarer Indikationsstellung und im Bewusstsein der potenziellen Risiken als Einzelheilversuch erfolgen. Dem verordnenden Arzt sollte bewusst sein, dass das zur Herstellung der Rezepturformulierung eingesetzte Fertigarzneimittel nicht für die Anwendung bei Kindern unter 15 kg Körpergewicht zugelassen ist und eine Aufklärungspflicht der Sorgeberechtigten über die damit verbundenen Risiken sowie über Therapiealternativen besteht. Diese Aufklärung sollte schriftlich dokumentiert und gegengezeichnet archiviert werden. Um eine sachgerechte Anwendung des Rezepturarzneimittels sicherzustellen, wird die Aushändigung einer schriftlichen Gebrauchsanweisung empfohlen.

## Fazit für die Praxis

- Die systemische Anwendung von Ivermectin bei Kindern mit einem Körpergewicht (KG) unter 15 kg entspricht einer Off-label-Therapie.
- Dennoch gibt es eine gute Datenlage, die eine Anwendung von Ivermectin in einer Dosierung von 200 µg/kgKG im Rahmen eines Einzelheilversuches bei rezidivierender Skabies rechtfertigt.
- Zur dosisgenauen Applikation wurde eine Rezepturformulierung als Ivermectin-Saft 400 µg/ml entwickelt und qualitätsvalidiert.
- Die 2‑malige Anwendung des Saftes im Abstand von 7 bis 10 Tagen zur Therapie der Skabies wurde mit ca. 120 € taxiert.
